# Adaptive Modifications of Maternal Hypothalamic-Pituitary-Adrenal Axis Activity during Lactation and Salsolinol as a New Player in this Phenomenon

**DOI:** 10.1155/2018/3786038

**Published:** 2018-04-10

**Authors:** Malgorzata Hasiec, Tomasz Misztal

**Affiliations:** Department of Animal Physiology, The Kielanowski Institute of Animal Physiology and Nutrition Polish Academy of Sciences, Instytucka 3, 05-110 Jablonna, Poland

## Abstract

Both basal and stress-induced secretory activities of the hypothalamic-pituitary-adrenal (HPA) axis are distinctly modified in lactating females. On the one hand, it aims to meet the physiological demands of the mother, and on the other hand, the appropriate and stable plasma cortisol level is one of the essential factors for the proper offspring development. Specific adaptations of HPA axis activity to lactation have been extensively studied in several animal species and humans, providing interesting data on the HPA axis plasticity mechanism. However, most of the data related to this phenomenon are derived from studies in rats. The purpose of this review is to highlight these adaptations, with a particular emphasis on stress reaction and differences that occur between species. Existing data on breastfeeding women are also included in several aspects. Finally, data from the experiments in sheep are presented, indicating a new regulatory factor of the HPA axis—salsolinol—which typical role was revealed in lactation. It is suggested that this dopamine derivative is involved in both maintaining basal and suppressing stress-induced HPA axis activities in lactating dams.

## 1. Introduction

During lactation, maternal anatomy, physiology, and behavior undergo considerable changes to ensure successful completion of the reproductive process—raising the offspring. Extensive growth of the mammary gland facilitates the synthesis and release of the required amount of milk. High milk production, resulting from intensive suckling, requires in turn high energy inputs, and therefore, fat reserves are utilized, as nutritional needs of the female increase. Ovulation inhibition, leading to periodic infertility, ensures sufficient time for nursing offspring. Moreover, mothers display crucial behavioral adaptations, such as protective behavior, aggression, and decreased anxiety [1]. Finally, considerable modifications of basal and stress-induced hypothalamic-pituitary-adrenal (HPA) axis activities are observed [2].

The present review focuses on the adaptive changes of HPA axis activity during lactation and possible mechanisms responsible for these modifications, mainly in the central nervous system (CNS). Most of the investigations have been performed in rats, but fragmentary data from other species clearly demonstrate intraspecific differences and are helpful to understand the physiology of breastfeeding women. Hence, the review highlights differences between rodents and nonrodent animals in this adaptation. Lastly, we also extend the current knowledge about new findings concerning the function of salsolinol, an endogenous dopamine (DA) derivative, in HPA axis activity in lactating sheep.

## 2. HPA Axis Activity

### 2.1. Basal Activity

Circulating adrenal corticosteroids are necessary to maintain metabolic homeostasis in the organism. Their concentration is tightly controlled by the CNS at the level of the hypothalamus and pituitary, which together with adrenals form the HPA axis. The hypothalamic regulatory site of the HPA axis is located within the paraventricular nucleus (PVN), which neurons release corticotropin-releasing hormone (CRH) and arginine vasopressin (AVP) into the hypophyseal portal circulation. They reach corticotropic pituitary cells via this route to stimulate adrenocorticotropin (ACTH) secretion [3]. The potency of action of these two neuropeptides may vary with species and physiological conditions. In rats and humans, AVP is a weaker ACTH secretagogue than CRH, but its action is essential for a full ACTH response when subjects are challenged by a variety of stressors [4]. However, studies performed on anestrous sheep provided contradictory data. Several reports indicated that AVP was a more potent stimulator of ACTH release than CRH [5–7]. It was demonstrated that the effects of CRH are likely mediated by the activation of protein kinase A, whereas the action of AVP is mainly mediated by the activation of protein kinase C [7]. It is possible that the interaction between these two pathways causes a variety of biochemical alterations within the corticotrophs, which are in turn responsible for the synergistic effect on ACTH. Other studies in sheep showed that an equimolar concentration of AVP and CRH infused into the third ventricle of the brain (IIIv) increased plasma cortisol concentration to the same extent [8, 9].

Regardless of the animal's lifestyle (day/night), HPA axis activity undergoes circadian alterations that consist of changes in pulsatile CRH, ACTH, and corticosterone/cortisol secretion [10–12]. The magnitude of the pulses appears to be the major variable in the pattern of this secretion over a 24 h period [13], although there is evidence that the sensitivity of corticotrophs to the negative feedback of corticosteroids also shows diurnal variation [14]. Circadian rhythmicity in pulsatile secretory activity of the HPA axis has been well demonstrated in rodents, ruminants, and primates, including humans, with peak levels occurring just before awakening [13–16]. According to such a pattern, the morning nadir and evening peaks of ACTH and corticosterone concentrations have been observed in rats, while both characteristics of the rhythm occur in the opposite periods in ruminants and humans.

Studies in rats have shown that basal HPA axis activity is distinctly increased in lactation due to a rise in nadir levels of corticosterone [17–20], while evening peak concentrations decrease [17, 21, 22], contributing to the flattening of the diurnal rhythm. Analogously, an increase in nadir levels of the hormone has been demonstrated for ACTH [21]. Such an increase in the secretory activity of the HPA axis is required to cope with the increased maternal metabolic demands, and on the other hand, its stability prevents neonatal exposure to varying glucocorticoid levels. Glucocorticoids have been demonstrated to freely enter maternal milk and affect offspring, causing long-term programming effects in postnatal and/or adult life [23, 24].

HPA axis activity during lactation is not modified in exactly the same manner in other species. In contrast to the findings in rats, no differences in basal HPA axis activity have been observed between lactating and nonlactating sheep and cows [25–27]. However, these studies did not examine changes over a 24-hour period. Studies performed in our laboratory also suggested the lack of differences in basal plasma ACTH and cortisol concentrations between midlactating and anestrous or postweaning sheep [28, 29]. Furthermore, a study on Japanese macaques (*Macaca fuscata*) showed that lactating females had even lower fecal glucocorticoid concentrations than cycling females during the mating season [30]. In women after parturition, the HPA axis gradually regains its prepregnancy state [31], although the precise time course of these changes has not been established. A higher basal level of cortisol was demonstrated in women 8 weeks postpartum compared to nonlactating ones [32]. On the other hand, Chiodera et al. [33] showed no differences in cortisol and ACTH concentrations in breastfeeding women 5–7 days after parturition compared to nonlactating subjects. Since cortisol levels fluctuate in the circadian rhythm, this discrepancy may result from different sampling times during the day. Therefore, it is necessary to precisely examine the time course to clarify the dynamics of changes in basal HPA axis activity in women during lactation.

### 2.2. Stress-Induced Activity

There is an increased activation of the HPA system in response to certain challenges requiring higher energy mobilization, for example, during stress response. However, numerous studies in lactating rats showed that stress-induced HPA axis activity is downregulated in response to many types of physical stressors, such as exposure to ether [20, 21, 34, 35], noise [36], electrical shock [35], and hypertonic saline [37] as well as immune challenges [38] and psychological stressors like the elevated plus-maze test and forced swimming [39]. This phenomenon is crucial for the aforementioned postnatal ontogenesis, since mother's glucocorticoids could be passed to the offspring with milk [40] and affect, for example, infant temperament [41]. Interestingly, the downregulation of a stress-induced HPA axis activity in lactating dams does not manifest in all situations. Dams exhibited greater ACTH and corticosterone responses when exposed to either predator odor or male intruder in the presence of the litter compared to analogically stressed dams in the absence of offspring [42]. Thus, when the stressor is a threat also to the offspring, the mother's HPA axis response to stress is much stronger. This indicates that a specific central mechanism “filters” environmental challenges, maintaining a reduced response of the mother's HPA axis to stress in the absence of a threat to pups, and allows an adequate neuroendocrine response when offspring is threatened [43].

Studies in sheep confirm hyporesponsiveness of the HPA axis in lactating dams. Lactating sheep displayed lower cortisol response to barking dogs [44, 45] as well as isolation and restraint stress [27] than nonlactating sheep. A critical role of the pups' presence in regard to hyporesponsiveness of the HPA axis during lactation was also demonstrated in this species. Interestingly, lactating ewes with lambs present showed a greater cortisol response to a barking dog than to isolation and restraint stress [45]. In our previous study, simultaneous isolation from the flock and lamb evoked higher ACTH and cortisol secretion in mothers than the isolation from the flock in the presence of their lamb [46]. The above observations suggest a greater HPA response to stressors when the safety of offspring is threatened. In another species, the common marmoset (*Callithrix jacchus*), lactating females maintained full HPA axis responsiveness to restrain stress when their infants were present [47]. Moreover, free-living lactating rhesus macaques (*Macaca mulatta*) had even higher cortisol levels than virgin females when they were captured with their offspring [48].

HPA axis response to stress in breastfeeding women also depends on the nature of stressors. Mothers during lactation had lower ACTH and cortisol responses to treadmill exercise in relation to nonlactating women [49], but cortisol response to CO_2_ inhalation was not affected in breastfeeding women compared with the control group [50]. Interestingly, hormonal response to social stress was reduced in breastfeeding women when tested 10 minutes after feeding their infants [51], but not after one hour of breastfeeding [52].

## 3. Mechanism of Adaptive Changes of the HPA Axis in Lactation

Most studies on modifications of HPA axis activity in lactating females were performed in rats and demonstrated that adaptive changes in the functioning of the HPA axis relate mainly to the hypothalamus and pituitary [2]. However, after dexamethasone treatment, an increase in plasma corticosterone level in response to exogenous ACTH was lower in the lactating group compared with controls [20], suggesting alterations also at the level of the adrenal gland.

Several adaptive changes of HPA axis activity result from modifications in the regulatory PVN region. It was found that mRNA transcript levels were reduced for CRH and greatly elevated for AVP within the PVN during lactation [19, 36, 38, 53]. Moreover, it was demonstrated that lactating rats exhibited a high degree of CRF and AVP colocalization in parvocellular PVN neurons, hypothalamic projections, and median eminence terminals compared to virgins [53]. The above data allow to assume that AVP plays a more important role in the regulation of basal ACTH secretion than CRH during lactation. Data concerning the potency of both ACTH secretagogues in lactating sheep are not available.

Adaptive changes within the anterior pituitary concern in particular lower sensitivity of corticotrophs to CRH. In lactating rats, treatment with CRH caused lower ACTH secretion than that observed in virgin rats [54]. Interestingly, separation of the litter for 48 h partially restored pituitary responsiveness to this neuropeptide. In contrast, the increased responsiveness of ACTH to AVP or the combination of AVP and CRH has been demonstrated in lactating rats [53, 54]. The precise mechanism of these alterations is not known, although it was demonstrated that the modified response to CRH was not the result of reduced CRH and AVP receptor levels in the pituitary gland [54]. Studies on women showed that the ACTH response to CRH treatment was blunted postpartum at 3 and 6 weeks, but not at 12 weeks [55].

Reduced response to stress in lactating females is also a consequence of a lower stimulation of parvocellular neurons within the PVN, as a marked decrease of stress-induced c-fos mRNA levels has been observed in these neurons in lactating rats [38, 56]. Moreover, a decrease in the stress-induced mRNA level of CRH [57] and AVP [38] has been shown within the PVN in lactating rats. Another study showed that the stress-induced AVP mRNA level in the parvocellular PVN was similar to that observed in virgin females [57]. However, this discrepancy might have resulted from differences in the stressors applied, that is, lipopolysaccharide injection versus immobilization.

Noradrenaline, which acts through *α*1-adrenergic receptors, is the primary excitatory neurotransmitter for the parvocellular PVN neurons in stressful situations [58]. Thus, HPA axis hyporesponsiveness to stress could be a consequence of a decreased noradrenergic input and/or lower sensitivity of the parvocellular PVN neurons to this neurotransmitter. Destruction of noradrenergic afferents terminating within the PVN in lactating rats did not reduce plasma ACTH concentration in lactating rats, as observed in virgin females [59]. In addition, central injection of *α*1- and *α*2-antagonists failed to inhibit ACTH secretory response to stress in lactating rats [60], while centrally administrated *α*1-agonist caused a significant elevation of plasma corticosterone concentration in virgin dams, but not in lactating ones [61]. On the other hand, stress-induced c-fos mRNA levels within the locus coeruleus did not differ between lactating and nonlactating rats [56].

Hyporesponsiveness of the HPA axis during lactation is not only a consequence of PVN modifications, but also a result of changes in other brain regions associated with HPA axis activity regulation. Reduced expression of c-fos mRNA was observed in the ventral part of the lateral septum and the medial amygdala after immobilization stress in lactating rats. It was, however, not found in the hippocampus, dentate gyrus, piriform cortex, locus coeruleus, dorsal vagal complex, and ventral tegmental area [56]. Interestingly, central administration of CRH activated neurons in the bed nucleus of the stria terminalis, lateral septum, medial and central amygdaloid nuclei, and PVN in virgin rats, but not in lactating counterparts [62]. Moreover, the basal level of CRH mRNA was increased in the dorsomedial region of the bed nucleus of stria terminals and decreased in the central nucleus of the amygdala during lactation [53]. Arriaga-Avila et al. [63] demonstrated that after exposure to immobilization stress, lactating rats have decreased DA and increased *γ*-aminobutyric acid (GABA) release in the medial prefrontal cortex region connected with, that is, the amygdala and hypothalamus.

Modifications within the glucocorticoid negative feedback could be one of the suspected reasons for the altered HPA axis function. However, the first research on this topic demonstrated contradictory results [20, 37, 64]. More recent data have shown that methylprednisolone, a synthetic corticosteroid, caused rapid attenuation of the HPA axis in virgin rats, but not in lactating ones [22]. Additionally, a study in humans demonstrated that dexamethasone had a lower ability to suppress plasma cortisol concentration in breastfeeding women [65]. The hippocampus is a promising research area on modifications within the negative feedback of the HPA axis during lactation. Numerous glucocorticoid receptors (GR) and mineralocorticoid receptors (MR) are present in this brain structure, signaling through both genomic and nongenomic pathways [66, 67]. Moreover, the hippocampus provides the GABAergic inhibitory network to the hypothalamus, especially to the PVN region [68, 69]. It has been shown that lactating animals have significantly reduced MR mRNA level within the hippocampus when compared with virgin animals [22] and that there is also a reduction in the GR density in the hippocampus during the first two weeks of lactation, as opposed to the hypothalamus or pituitary [64]. Considering that PVN CRH neurons are subject to robust tonic inhibition mediated by GABAergic inputs [69], it is likely that the limited corticosteroid signaling in the hippocampus of lactating females increases the basal activity of the HPA axis. On the other hand, the GABAergic system was shown to be upregulated in lactating rats in the presence of pups based on measurements carried out in the cerebrospinal fluid [70].

The hippocampus is also one of the most plastic regions in the adult brain, since its neurons undergo continuous remodeling throughout the lifespan [71]. It has consistently been demonstrated in rats and sheep that cell proliferation in the maternal hippocampus is reduced during the early postpartum period compared to age-matched virgin females [72–76]. This phenomenon occurs simultaneously with an elevation in basal glucocorticoid level during lactation [72], and both are abolished by weaning [73]. The inhibition of cell proliferation is more likely to be a consequence of hormonal changes that occur at parturition, since sheep mothers, separated from their newborn lambs immediately after parturition, express a similar decrease in cell proliferation as those staying with their lambs [72, 75]. The relationship between hippocampal neurogenesis and hyporesponsiveness of the HPA axis during lactation has not yet been fully explored.

## 4. Factors Involved in The Modulation of HPA Axis Activity during Lactation

### 4.1. Suckling

Walker et al. [21] suggested that increased basal HPA axis activity in lactating rats was not caused by the caloric drain due to milk production. In light of this, other stimuli associated with the presence of the offspring, like suckling by the infant, seem to be crucial for the formation of mother's HPA axis adaptations. In rats, separation of pups from lactating mothers caused the return of basal ACTH and corticosterone concentrations to the levels observed in nonlactating females [19, 21]. Moreover, mother-offspring reunion after a 4-hour separation resulted in a raise in maternal plasma ACTH and cortisol concentrations [21]. More detailed data were provided by a study using dynamic blood sampling in rats for 24 hours. Three days after weaning, on day 10 of lactation, basal HPA activity was substantially suppressed, which was manifested by a reduced basal level of corticosterone and its pulse number as well as delayed diurnal increase of this hormone [22]. Two weeks after pups' weaning, the mean corticosterone concentration and pulse characteristic returned to the level observed in the virgin control, although the delayed diurnal rise phase was still observed [22].

The suckling stimulus is also crucial for maintaining a reduced stress response in lactating animals. Separation of the litter from lactating dams for 48 hours restored ACTH response to CRH treatment in mothers [54]. The paramount importance of suckling in maintaining HPA axis hyporesponsiveness has been confirmed in a study on lactating rats, whose nipples were surgically removed [34]. Although these females remained with the offspring, it was insufficient to reduce ACTH and corticosterone stress response, as opposed to lactating dams with intact nipples. Moreover, suckling by pups induced an increase in c-Fos expression in many maternal brain areas, including the locus coeruleus, which was not observed to the same extent when rats were exposed to their pups that were prevented from suckling by a wire screen [77]. Research in sheep also provided valuable data on HPA axis activity in lactating females. It was demonstrated that lactating sheep had a lower response to isolation and restraint stress than nonlactating controls [27]. The greatest reduction of cortisol response occurred in lactating ewes with lambs present and able to suckle, lower in lactating ewes with lamb present but unable to suckle, and the lowest in mothers isolated from offspring for 16 hours before and during the experiment [27]. Our study also confirmed that suckling reduced the stress-induced increase in plasma ACTH and cortisol concentrations in lactating sheep [46]. We observed that lactating sheep isolated from the flock had lower plasma ACTH and cortisol concentrations when sucked by offspring during the isolation [46]. These studies have clearly indicated the importance of suckling, although stimuli associated with the presence of pups are also essential. In contrast to animals, reduction of the HPA axis response to a psychosocial stressor in women seemed to occur only shortly after breastfeeding, but not constantly during lactation [78].

The direct impact of suckling on the HPA axis in lactating animals raises the question about the mechanism of this phenomenon. Since suckling caused a substantial release of prolactin and oxytocin, which also act in the brain and are widely involved in maternal adaptations during lactation [1], these both hormones may be considered as factors mediating the suckling effect on HPA axis activity in lactating females.

### 4.2. Prolactin

Prolactin plays a crucial role in the onset of maternal behavior [79, 80] and exerts an anxiolytic effect in both lactating and nonlactating rats [81]. This hormone has also been documented as having inhibitory properties against HPA axis responses to stress in virgin and lactating rats [81].

The HPA axis can be regulated by prolactin originating from two sources: synthesized by lactotrophs in the anterior pituitary and released into the peripheral circulation, or synthesized directly within the hypothalamus [80, 82, 83]. Suckling increases prolactin secretion during lactation not only within the anterior pituitary, but also within the PVN and medial preoptic area [84, 85]. Chronic infusions of antisense oligonucleotides against the long isoform of the prolactin receptor caused increases in stress-induced ACTH release in lactating rats, confirming that endogenous prolactin was indeed involved in the inhibition of HPA axis stress response during lactation [80]. Moreover, infusions of prolactin into the PVN suppressed cortisol response to stress in both lactating and nonlactating sheep [44].

The action of prolactin within the CNS is possible through its receptors present in the brain, especially within the choroid plexus and hypothalamus [86–88]. Expression of prolactin receptors during lactation in rats increases within the medial preoptic nucleus, periventricular nucleus, and arcuate nucleus. Moreover, prolactin receptor immunoreactivity or mRNA for prolactin receptors is detected within the PVN, ventromedial hypothalamic nucleus, and supraoptic nucleus only during lactation, but not in diestrous females [89]. On the other hand, Blume et al. [90] have suggested that prolactin inhibits HPA axis activity indirectly through the modulation of afferent inputs to the PVN.

### 4.3. Oxytocin

Oxytocin is the second hormone abundantly released following suckling. Although peripheral oxytocin, originating from the posterior pituitary, poorly penetrates the CNS [91], suckling induces oxytocin release within the SON and PVN [92, 93]. Moreover, oxytocin immunoreactivity increases during lactation not only within the PVN and SON, but also in the bed nucleus of the stria terminalis, the periventricular part of the medial preoptic, anterior commissural nuclei in sheep [94], and in the brainstem and the ventral septum in rats [95]. Oxytocin receptors are also widely distributed in the CNS [96], and oxytocin binding within the hypothalamus is increased in lactating females [97]. The inhibitory effect of oxytocin on the HPA axis was suggested by studies, in which oxytocin was infused intracerebroventricularly in ovariectomized, estradiol-treated rats [98, 99]. The latter authors have shown reduced corticosterone response to noise and restraint stress. Moreover, it has been demonstrated that restraint stress does not increase c-fos mRNA level within the dorsal hippocampus, ventrolateral septum, and PVN in animals treated with oxytocin, as observed in control animals [99]. However, intracerebroventricular treatment with oxytocin antagonist increased the stress-induced plasma ACTH and corticosterone concentration in virgin females, but not in lactating ones, demonstrating the inhibitory effect of endogenous oxytocin on stress-induced HPA axis activity only in virgin rats [100]. In sheep, oxytocin infusion into the posterior pituitary reduced cortisol response to barking dog in both lactating and nonlactating females, confirming the oxytocin inhibitory effect on stress-induced HPA axis activity in mammals [44].

## 5. Salsolinol: A New Putative Inhibitor of HPA Axis Activity during Lactation

Emphasizing the importance of suckling in the inhibition of stress-induced HPA axis activity, it is worth considering whether other factors, especially of neuronal origin, mediate its action. This drew our attention to salsolinol, an endogenous DA derivative [101], which is abundantly released within the infundibular nucleus/median eminence (IN/ME) of lactating sheep [102].

Salsolinol (1-methyl-6,7-dihydroxy-1,2,3,4-tetrahydroisoquinoline) is a product of DA and acetaldehyde condensation resulting from the enzymatic or nonenzymatic Pictet-Spengler reaction [103]. The existence of salsolinol synthase has been proposed [104], but it is neither fully characterized nor its amino acid sequence is determined. The activity of this enzyme and/or high concentrations of salsolinol were found in several brain regions, mostly rich in dopaminergic neurons of rats, ruminants, and humans [102, 104–106]; salsolinol was also found in the median eminence as well as in the neurointermediate (NIL) and posterior lobes of the pituitary [107, 108].

In the past decades, salsolinol was considered as a compound with a negative impact on the CNS. It was associated with some neurobiological effects of ethanol [109] and was also suspected of involvement in the etiopathogenesis of Parkinson's disease [103]. In addition to the involvement in pathological processes, salsolinol may act as a neuromodulator of some physiological functions in the CNS. It is considered as a putative hypothalamic prolactin-releasing factor in rodents [107] and ruminants [84, 108], in some specific conditions, especially during the suckling stimulus [102, 110, 111]. Toth et al. [107] found that salsolinol concentration in NIL of lactating rats revealed parallel increases with plasma prolactin in response to a brief suckling stimuli, following 4 h separation. They also demonstrated that salsolinol can elevate prolactin release in pituitary cell cultures as well as in hypophysectomized rats bearing anterior lobe transplants under the kidney capsule. In lactating sheep, suckling induced an increase in the extracellular concentration of salsolinol within the IN/ME, which was closely related to the changes in plasma concentration of prolactin [102]. Moreover, salsolinol effectively stimulated both prolactin and oxytocin secretion in lactating sheep, when infused into the IIIv [84, 112]. Furthermore, intracerebroventricular infusions of 1-MeDIQ, a compound antagonizing the salsolinol actions, attenuated both prolactin [111] and oxytocin [113] surge evoked by the suckling stimulus, suggesting that salsolinol might mediate suckling-induced release of both hormones.

In light of the above reports, it was important to investigate whether salsolinol participates in the inhibition of HPA axis activity during lactation. Several studies performed in our laboratory during the last few years aimed at investigating this assumption. Initially, it was demonstrated that intracerebroventricular infusions of salsolinol inhibited the increase in plasma ACTH and cortisol levels in lactating sheep induced by isolation stress [46]. Similar infusions, but 48 hours after weaning of 8-week-old lambs, also inhibited ACTH and cortisol response to handling stress [28]. The results of both studies suggested the inhibitory potency of salsolinol with respect to the HPA axis. Surprisingly, salsolinol infusions had no effect on CRH concentration in perfusates from the IN/ME. Furthermore, isolation stress also did not affect CRH concentrations in perfusates from this site [46], although this could be justified by a long-term collection of one perfusate. On the other hand, the lack of changes in CRH release within the ovine IN/ME, following the exposure to stress with simultaneous increase in plasma ACTH and cortisol concentrations, suggests that, as in rats, AVP is an important ACTH-releasing factor during lactation. However, to the best of our knowledge, no direct data on this topic is available for lactating sheep. Analogously to the above, both suckling and salsolinol decreased ACTH and cortisol response to stress, but did not change CRH concentration [46], which again was indicated on AVP neurons, through which salsolinol could affect ACTH secretion. However, when 1-MeDIQ (a compound antagonizing salsolinol actions) was infused into the IIIv in stressed lactating sheep, the animals exhibited substantially higher concentrations of perfusate CRH and plasma cortisol than stressed animals treated with vehicle infusions [46]. This suggests that endogenous salsolinol is indeed an inhibiting factor of stress-induced CRH release in lactating ewes, although its effect on AVP neurons remains to be clarified. According to our findings, the influence of salsolinol on stress response in lactating sheep was similar to the change caused by suckling, pointing to salsolinol as a factor mediating the effect of suckling on the HPA axis [46]. Interestingly, this effect of salsolinol in lactating sheep is specific not only for stress-induced HPA axis activity, but also for the basal activity of this neurohormonal axis [28]. Considering that the basal activity of the HPA axis in lactating sheep is unaltered compared to nonlactating sheep [25, 27], it appears that the salsolinol effect related to basal HPA axis activity is counteracted by other stimulating factors in physiological conditions.

Knowledge about the detailed mechanism of salsolinol action in various physiological processes is still limited. Specific and saturable binding sites for this compound were found in the striatum, cortex, hypothalamus, median eminence, and in the NIL of the pituitary [114]. It was suggested that salsolinol binding is closely related to a site that can also recognize and use DA as a signaling molecule, but its properties differ from any known DA receptors. Until now, however, the structure of the salsolinol-specific receptor has not been recognized. Previous studies on the physiological effects of salsolinol on the CNS pointed to decreases in catecholamine levels [115, 116]. This is consistent with the ability of salsolinol to decrease tyrosine hydroxylase (TH) activity, an enzyme limiting the rate of catecholamine synthesis [117, 118].

It is difficult to explain the mechanism of salsolinol effect on the HPA axis due to the lack of knowledge about the detailed mechanism of salsolinol action within the CNS. An interaction with the central noradrenergic system is one of the possible routes, and the main premise is that salsolinol suppresses TH activity [117, 118]. It was shown that salsolinol, similarly to suckling, significantly attenuated stress-induced noradrenaline increases within the intracellular space of the IN/ME, confirming the inhibitory effect of salsolinol on the noradrenergic system [46]. In addition to changes in noradrenaline, we observed a considerable increase in DA concentration within the IN/ME during stress response, and both salsolinol and suckling greatly diminished this reaction [46]. Moreover, our earlier study demonstrated that salsolinol infusion into the IIIv of unstressed, anestrous sheep reduced DA concentration within the IN/ME to an undetectable level [116]. Although the regulation of the HPA axis by DA has not been widely studied, it is suspected that this catecholamine could stimulate secretory activity of the HPA axis [119–121]. The suppressed dopaminergic system could, therefore, contribute to the decreasing HPA axis response to stress. However, it should be noted that the measurements of noradrenaline and DA in our study refer to the IN/ME and may not entirely reflect the amounts of these neurotransmitters within the PVN, and thus further research focusing directly on the PVN is necessary to confirm our presumptions.

It is believed that salsolinol is a prolactin-releasing factor; hence, one can expect that the inhibition of HPA axis activity by this compound could result from the increased prolactin release. However, despite the inhibition of ACTH and cortisol stress response, salsolinol does not increase peripheral prolactin concentration in stressed-lactating sheep, suggesting that prolactin does not mediate this salsolinol effect [46]. In order to verify this observation, we conducted serial salsolinol intracerebroventricular injections in anestrous sheep subjected to handling stress at the turn of winter and spring, when prolactin secretion was significantly reduced [13]. As a result of salsolinol injections, sheep had lower ACTH and cortisol response to stress compared to vehicle-treated animals, but there were no changes in plasma prolactin concentration, confirming that salsolinol action in regard to the HPA axis occurred without prolactin mediation [29]. Oxytocin should also be considered as a potential mediator of the discussed phenomenon, since salsolinol can mediate suckling-induced release of this hormone in lactating sheep [113]; however, it still remains to be investigated. Moreover, it requires a detailed examination of the relationship between prolactin and oxytocin and particularly which of these hormones responds first to salsolinol. This topic, however, is beyond the scope of this review.

## 6. Perspectives

Abnormalities in the functioning of the HPA axis in women are often correlated with postpartum depression [122, 123]. Therefore, understanding HPA axis adaptive mechanisms in lactating females seems to be crucial for the prevention of this disorder. Particular attention should be paid to suckling, because some articles have reported that nonbreastfeeding women have a higher level of depressive symptoms compared to breastfeeding women, although antidepressant effects of breastfeeding have still not been proven [124]. Despite the practical needs of medicine, and the number of clinical and basic studies on the functioning of the HPA axis during lactation, the issue has not yet been fully investigated. Our research on the role of salsolinol in this phenomenon provides new and intriguing data and also initiates subsequent questions. In particular, they include the following: Does the inhibitory effect of salsolinol on the HPA axis occur in lactating rats—the main model for studying the mechanism of HPA axis adaptation during lactation? How does salsolinol regulate AVP and CRH secretion within the PVN and whether the noradrenergic system is involved? Does oxytocin mediate its effect on the HPA axis? The proper mechanism of salsolinol action in the CNS and, especially, the specific salsolinol receptor structure is still the great unknown. In addition to the use of other animal models, there is also a need to conduct *in vitro* studies to analyze the possibility of direct salsolinol action on the pituitary. Despite these several unknowns, our research provides data suggesting a new role of salsolinol in the physiology of lactation, being the next piece of the puzzle.
